# Effect of different surgical approaches on the prognosis of patients with postoperative radiotherapy for stage IIB–IVA esophageal squamous cancer

**DOI:** 10.1186/s12957-022-02739-3

**Published:** 2022-08-30

**Authors:** Mingcheng Gao, Yulin Zhu, Yan Gu, Zhan Shi, Jixiang Wu, Huiwen Chang, Jianxiang Song

**Affiliations:** Department of Cardiothoracic Surgery, Yancheng Third People’s Hospital, The Sixth Affiliated Hospital of Nantong University, The Yancheng School of Clinical Medicine of Nanjing Medical University, 2 Xindu West Road, Yancheng, 224000 Jiangsu China

**Keywords:** Esophageal squamous cell carcinoma, Surgical approach, Postoperative adjuvant therapy, Radiotherapy, Survival analysis

## Abstract

**Objective:**

To investigate the effect and clinical significance of different thoracic surgical approaches for patients with stage IIB–IVA esophageal squamous cell carcinoma on the survival and prognosis of postoperative radiotherapy patients.

**Methods:**

One hundred thirty-two patients with stage IIB–IVA esophageal squamous cancer who received radiotherapy after surgery were screened for baseline characteristics and survival analysis. The Kaplan-Meier method was used to draw the survival curve for the follow-up data, and the log-rank test was used to compare the difference in survival rate between the two groups. The Cox regression model was used for multivariate survival analysis.

**Result:**

For stage IIB–IVA esophageal squamous cell carcinoma, the results of multivariate analysis showed that different surgical methods and clinical staging were independent factors affecting the survival and prognosis of patients after radiotherapy. The 1-, 3-, and 5-year survival rates of patients with advanced esophageal cancer through the left chest approach were 84.2%, 61.4%, and 36.8% respectively. The 1-, 3-, and 5-year survival rates of patients with advanced esophageal cancer through the right chest approach were 73.3%, 40.0%, and 21.3% respectively. There was no significant difference in the 1-year survival rate (*P* = 0.135) between the two surgical procedures. The 3-year survival rate (*P* < 0.05) and the 5-year survival rate (*P* < 0.05) were significantly different.

**Conclusion:**

For patients with stage IIB–IVA esophageal squamous cell carcinoma undergoing radiotherapy after surgery, the long-term survival prognosis of patients after the left thoracic approach is significantly higher than that of the right thoracic approach.

## Introduction

Esophageal cancer is one of the most common malignant tumors in the world [1]. Due to the complex anatomical structure of the esophagus and numerous lymph nodes involved in removal, the rate of postoperative recurrence and metastasis after surgery is high [2, 3]. Different surgical methods, neoadjuvant therapy and postoperative adjuvant therapy have a great influence on the prognosis of patients [4–7]. Radiation therapy can be used as an important adjuvant therapy for patients with esophageal cancer after surgery. However, it is accompanied by adverse reactions caused by radiotherapy, including the radiation reaction of radiation to the thoracic remnant stomach, which seriously affects the prognosis of patients [8].

Different surgical approaches due to the different positions of the thoracic stomach often lead to differences in the effect of postoperative radiotherapy. At present, there are few relevant literatures on the effect of different surgical methods on the prognosis of postoperative radiotherapy patients. Despite of the advantages of right thoracic approach in lymph node dissection [9], the left thoracic approach can provide a more favorable environment for postoperative radiotherapy.

This study retrospectively analyzed the survival of 132 patients with stage IIB–IVA esophageal squamous cell carcinoma who underwent left thoracic and right thoracic approaches followed by postoperative radiotherapy. We compared survival outcomes in radiotherapy patients with different thoracic surgical approaches, in order to provide clinical evidence for the optimal surgical approach and postoperative radiotherapy for patients with esophageal cancer.

## Materials and methods

### General clinical data

Patients with esophageal cancer who received surgical treatment in Affiliated Hospital 6 of Nantong University from 2013 to 2015 were retrospectively collected, and 151 patients with esophageal cancer who had clinical and pathological stages IIB–IVA and received postoperative radiotherapy were screened. Among 151 patients, 18 cases were excluded from follow-up, and 1 case was due to other reasons. Finally, a total of 132 patients were enrolled, including 84 males and 48 females, with an average age of 65.3 ± 7.3 years. All patients were confirmed to be esophageal squamous cell carcinoma by clinicopathological examination. According to different surgical methods, they were divided into left thoracic approach group (*n* = 57) and right thoracic approach group (*n* = 75). All clinical and pathological staging were based on the eighth edition of the TNM staging system [10], including 8 cases of T1N1M0 stage, 1 case of T1N2M0 stage, 8 cases of T2N1M0 stage, 2 cases of T2N2M0 stage, 61 cases of T3N0M0 stage, 24 cases of T3N1M0 stage, and T3N2M0 stage 18 cases, 10 cases of T3N3M0 stage; 69 cases of stage IIB, 9 cases of stage IIIA, 44 cases of stage IIIB, and 10 cases of stage IVA.

### Inclusion and exclusion criteria

The inclusion criteria were that all patients did not receive neoadjuvant chemoradiotherapy before surgery, the postoperative pathological TNM staging reached IIB–IVA stage, all pathological types were squamous cell carcinoma, and postoperative preventive radiotherapy was given according to the standard treatment.

The exclusion criteria were as follows: distant metastases found before surgery; positive surgical margins; lack of relevant pathological data during the investigation and follow-up; patients lost to follow-up or refusing to cooperate with the investigation; and patients who died of other causes other than esophageal cancer (such as a car accident).

### Surgical methods and postoperative radiotherapy

#### Left thoracic approach

The patient took the right side, made an incision on the posterolateral side of the left chest, and entered the chest layer by layer from the sixth intercostal space. Free the thoracic esophagus and dissect the thoracic lymph nodes. Then, we dissociated the stomach through the diaphragm, paying attention to preserving the vascular arch of the greater curvature of the stomach, dissected the abdominal lymph nodes, and elevated the remnant stomach to the left chest. Finally, a mechanical esophagus-gastric anastomosis was performed on the top of the chest. After surgery, the remnant stomach was located in the left thoracic cavity.

#### Right thoracic approach

The patient was placed in the left lateral decubitus position. We made an incision on the anterolateral side of the patient's right thoracic fifth intercostal space and entered the thoracic cavity. We dissociated the thoracic esophagus and dissected the surrounding lymph nodes. Afterwards, we adjusted the patient's position to supine position, took the upper abdominal incision, dissected the abdominal lymph nodes, dissociated the stomach, and cut the stomach into a tubular stomach with a stapler. Finally, we made an oblique incision on the right side of the neck, freed the cervical esophagus, dissected the cervical lymph nodes, removed most of the esophagus, lifted the tubular stomach to the neck, and anastomosed at the right neck. After the operation, the residual stomach was located in the posterior mediastinum.

#### Postoperative radiotherapy

We adopted sIMRT (static intensity modulated radiotherapy) technology in this study. The clinician outlined the radiotherapy target area under the guidance of CT, including the original tumor bed area, anastomotic stoma and corresponding lymphatic drainage area (bilateral supraclavicular area, mediastinum ± upper abdominal cavity corresponding lymphatic drainage area). The dose was 50/50.4 Gy, a single dose of 1.8/2.0 Gy, 5 times/week.

### Follow-up

The survival time was calculated from the day of surgery to the time of death. The follow-up data of all cases were complete, and the deadline for data collection was December 31, 2020. Overall survival (OS) was defined as the time from the day of surgery to the date of death or termination of follow-up.

### Statistical analysis

All data were carried out by SPSS 22.0 software package, and GraphPad Prism 7.0 was used for graphing. Data collection, verification and calculation were carried out by two people. The enumeration data were used as relative numbers, and the *χ*^*2*^ test was used to compare the differences between the groups. The follow-up data were drawn by the Kaplan-Meier method, and the log-rank test was used to compare the survival rate differences between the two groups. Univariate and multivariate analyses were performed using COX proportional hazards model, and *P* < 0.05 was considered statistically significant.

## Result

### Case data

There were 132 patients in the whole group. The deadline for follow-up was December 2020. The survival situation of the whole group is shown in Table 1.

**Table 1:** General information of 132 patients with advanced esophageal cancer (*n*)

Variable	Left thoracic approach(*n* = 57)		Right thoracic approach (*n* = 75)		*χ*^*2*^	*P*
	Patients	Incidence(%)	Patients	Incidence(%)		
Sex					0.689	0.406
Male	34	59.6	50	66.7		
Female	23	40.4	25	33.3		
Age					0.386	0.534
< 60	9	15.8	15	20.0		
≥ 60	48	84.2	60	80.0		
T stage					0.766	0.682
T1	4	7.0	5	6.8		
T2	3	5.3	7	7.6		
T3	50	87.7	63	85.6		
N stage					7.014	0.071
N0	33	57.9	28	37.3		
N1	11	19.3	29	38.7		
N2	9	15.8	12	16.0		
N3	4	7.0	6	8.0		
Clinical stage					5.656	0.130
IIB	36	63.2	33	44.0		
IIIA	4	7.0	5	6.7		
IIIB	13	22.8	31	41.3		
IV	4	7.0	6	8.0		

### Survival analysis

For stage IIB–IVA esophageal cancer, the 1-year survival rates of the left thoracic approach and the right thoracic approach were 84.2% and 73.3% respectively. The 3-year survival rates were 61.4% and 40.0% respectively. The 5-year survival rates were 36.8% and 21.3% respectively. There was no significant difference in the 1-year survival rate between the two procedures (*χ2* = 2.235, *P* > 0.05).

**Fig. 1:**
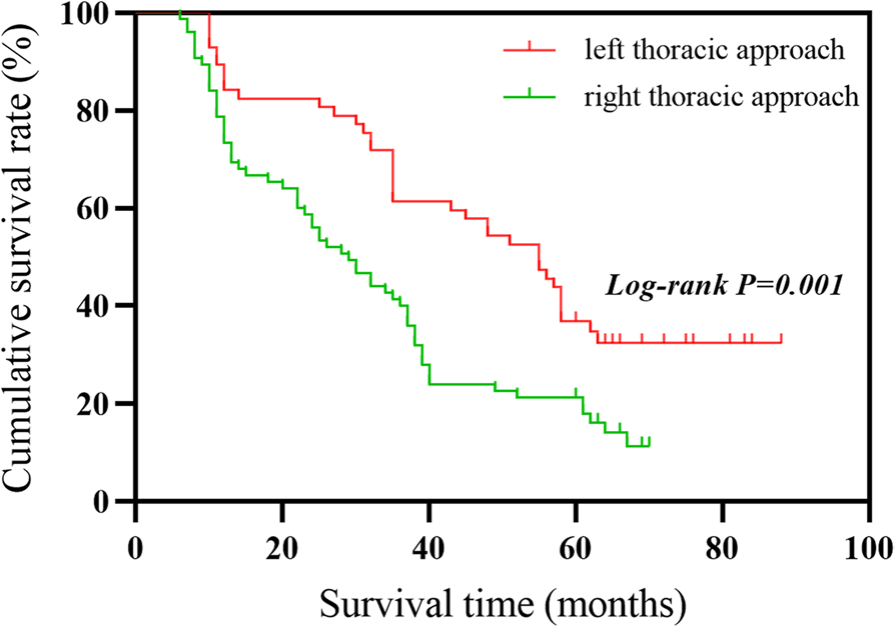
KM survival curve

There were significant differences in the 3-year survival rate (*χ2* = 5.936, *P* < 0.05) and the 5-year survival rate (*χ2* = 3.861, *P* < 0.05) between the two procedures. We used the Kaplan-Meier method to plot survival curves for follow-up data and used the log-rank test to compare the difference in survival rate between the two groups. The survival curve is shown in Fig. 1. The log-rank test showed that the OS of the left thoracic approach was longer than that of the right thoracic approach (*χ2* = 11.482, *P* < 0.01), and the difference was statistically.

### Univariate and multivariate COX proportional hazards regression model analysis on the survival and prognosis of patients with stage IIB–IVA esophageal cancer

Taking the patient's survival time as the dependent variable, and taking gender, age, T stage, N stage, clinical stage, and surgical method as the independent variables, the univariate COX regression model analysis showed that: for stage IIB–IVA esophageal cancer, N stage, clinical stage, The surgical method was an influential factor affecting the survival and prognosis of patients, and the difference was statistically significant (*P* < 0.05), as shown in Table 2. The indicators with statistically significant differences in univariate analysis (N stage, clinical stage, and surgical method) were used as independent variables, and the patient survival time was used as the dependent variable. After excluding confounding factors, multivariate COX regression model analysis showed that different surgical methods (HR 1.527, 95% CI 1.012–2.303, *P* = 0.044), clinical stages (HR 1.970, 95% CI 1.311–2.958, *P* = 0.001) were independent factors affecting the survival and prognosis of patients after postoperative radiotherapy. The OS of patients after left thoracic approach was significantly higher than that of right thoracic approach (*P* < 0.05), see Table 3 for details.

**Table 2:** Univariate COX proportional hazard regression model analysis of the prognostic factors of patients with esophageal squamous cell carcinoma

Variable	*β*	SE	Wald	*P*	HR	*95%CI*
Sex	0.182	0.203	0.800	0.371	1.199	0.805 ~ 1.787
Age	0.145	0.266	0.295	0.587	1.156	0.686 ~ 1.948
T stage	0.194	0.192	1.026	0.311	1.214	0.834 ~ 1.769
N stage	0.768	0.108	50.860	0.000	2.156	1.746 ~ 2.664
Clinical stage	0.782	0.102	59.113	0.000	2.185	1.790 ~ 2.667
Surgical approach	0.677	0.207	10.720	0.001	1.967	1.312 ~ 2.950

**Table 3:** Multivariate COX proportional hazards regression model analysis of factors influencing the poor prognosis of patients with esophageal squamous cell carcinoma

Variable	*β*	SE	Wald	*P*	HR	95%CI
N stage	0.082	0.227	0.131	0.717	1.085	0.696~1.692
Clinical stage	0.678	0.207	10.672	0.001	1.970	1.311~2.958
Surgical approach	0.423	0.210	4.065	0.044	1.527	1.012~2.303

## Discussion

Esophageal cancer is one of the leading causes of cancer morbidity and mortality in the world [11, 12]. In China, the top 5 leading causes of cancer death among both men and women were lung, bronchial, gastric, liver, esophagus, and colorectal cancers successively, and esophageal cancer ranks fourth [13]. Recent studies have shown that for patients with advanced esophageal cancer, recurrence or distant metastasis occurs in more than half of patients after surgery alone [14, 15]. And chemotherapy, radiotherapy, and targeted drug therapy, as important adjuvant therapy after esophageal cancer, have attracted more and more attention [16–18]. According to the Chinese 2021 Guidelines for Radiation Therapy for Esophageal Cancer, postoperative radiotherapy is recommended for lymph node-positive, pT3-4aN0 stage esophageal cancer, and high-risk pT2N0 adenocarcinoma [19].

At present, there are two main types of radical resections for esophageal cancer: the left thoracic approach and the right thoracic approach. Compared with the right thoracic approach, due to the obstruction of the left thoracic aortic arch and the narrowness of the upper arch triangle during the operation, the upper mediastinal lymph node dissection is not complete in the left thoracic approach [20]. However, the left thoracic approach stretches the remnant stomach to the left thoracic cavity, while the right thoracic approach stretches the remnant stomach to the posterior mediastinum. This creates favorable conditions for postoperative radiotherapy. Then radiotherapy after the left thoracic approach can keep the remnant stomach far away from the radiation target area of the original esophageal bed lymphatic drainage area, reducing the radiation exposure to the remnant stomach. It can also allow better exposure of the target area for lymphatic drainage of the original esophageal bed, so as to effectively ensure the effect of radiotherapy and improve the prognosis of patients.

The purpose of this study was to compare the effects of left-thoracic approach and right-thoracic approach on the survival rate of postoperative radiotherapy in patients with stage IIB–IVA esophageal squamous cell carcinoma. The 132 standard patients were included in this study and they were all treated with standard postoperative radiotherapy. The 1-, 3-, and 5-year survival rates of patients undergoing radiotherapy for left thoracic approach were 84.2%, 61.4%, and 36.8%. The 1-, 3-, and 5-year survival rates of patients with postoperative radiotherapy were 73.3%, 40.0%, and 21.3%. There was no significant difference in the 1-year survival rate (*P* = 0.135) between the two approaches. However, there were significant differences in the 3-year survival rate (*P* < 0.05) and the 5-year survival rate (*P* < 0.05). This suggests that the two surgical methods have significant differences in the long-term survival rate of patients. The results show that for patients with stage IIB–IVA esophageal squamous cell cancer, the survival time of patients with postoperative radiotherapy in the left thoracic approach is significantly higher than that in the right thoracic approach.

Although right thoracic esophagectomy is more effective than left thoracic esophagectomy for lymph node dissection for esophageal cancer [21–23], there is no prospective study evidence whether patients really benefit from radical lymph node dissection and the optimal extent of lymph node removal during esophagectomy still remains unclear [24, 25]. Two studies from Sweden and the United Kingdom showed that intraoperative lymph node dissection for esophageal cancer had no significant effect on the survival and prognosis of patients with esophageal cancer [26, 27].

Radiotherapy, as a supplementary treatment for esophageal cancer after surgery, can inhibit the further spread of the tumor and improve the survival rate of patients. Researches have shown that postoperative radiotherapy, especially for those patients with positive postoperative pathological lymph nodes, can reduce postoperative lymph node recurrence and improve patient survival time [28, 29]. However, extensive radiation often causes severe systemic reactions, especially the irradiation of the thoracic remnant stomach. In severe cases, it can lead to complications such as gastric bleeding and perforation. Many patients with poor tolerance even need to reduce the radiation dose or discontinue radiation [30, 31]. It is well known that the occurrence of radiation gastritis after esophageal cancer surgery is closely related to the location of the thoracic stomach. With the introduction of thoracoscopy, for esophageal cancer patients with conventional right thoracic approach, the postoperative residual stomach is mostly located in the posterior mediastinum. This just located within the target area of the lymphatic drainage area in the esophageal tumor bed area and leads to a large range and dose of radiation to the remnant stomach, which eventually cause to an increase in the incidence of radiation gastritis. The higher the dose, the higher the incidence of radiation gastritis [32]. Song Chunyang et al. [33] studied 104 patients with esophageal cancer who underwent postoperative intensity-modulated radiotherapy (IMRT), and the incidences of grade ≥ 2 acute radiation thoracic gastritis in the posterior mediastinal thoracic stomach group and the left thoracic stomach group were respectively 69.23% and 16.92%. The probability of postoperative radiation gastritis in patients with right thoracic approach was significantly higher than that with left thoracic approach, which greatly affected the prognosis of patients. At the same time, due to the blocking of rays by the posterior mediastinal remnant stomach, radiotherapy cannot give sufficient radiation dose to the postoperative esophageal tumor bed area and lymphatic drainage area, thus affecting the prognosis of patients [34]. In conclusion, it is recommended that multidisciplinary consultation should be conducted before radical resection of esophageal cancer. According to factors such as preoperative clinical stage and neoadjuvant therapy, the surgical method should be determined to create conditions for postoperative radiotherapy.

In summary, for patients with stage IIB–IVA esophageal squamous cell carcinoma, the long-term survival rate of postoperative radiotherapy in the left thoracic approach is higher than that in the right thoracic approach. Therefore, this study considers that for patients with esophageal squamous cell cancer who need supplemental radiotherapy after surgery, a left thoracic approach can be added to the right thoracic approach during surgery to guide the remnant stomach from the posterior mediastinum to the left thoracic cavity. This not only achieves the effect of minimally invasive treatment, but also reduces postoperative complications such as radiation gastritis, and improves the survival prognosis of patients. Due to irresistible factors such as loss to follow-up during the follow-up process of patients, further prospective randomized controlled trials are needed to further validate this study.

## Conclusion

In conclusion, we compared different surgical approaches in patients with esophageal cancer undergoing radiotherapy by constructing a survival analysis model. For patients with stage IIB–IVA esophageal squamous cell carcinoma undergoing radiotherapy after surgery, the long-term survival prognosis of patients after the left thoracic approach is significantly higher than that of the right thoracic approach.

## Data Availability

The datasets used and/or analyzed during the current study are available from the corresponding author on reasonable request.

## Abbreviations

OS: Overall survival
sIMRT: Static intensity modulated radiotherapy
IMRT: Intensity-modulated radiotherapy
